# Regulation of the small GTPase Ran by miR-802 modulates proliferation and metastasis in colorectal cancer cells

**DOI:** 10.1038/s41416-020-0809-7

**Published:** 2020-03-25

**Authors:** Xin Wang, Danxiu Li, Lina Sun, Gaofei Shen, Hao Liu, Hao Guo, Minghui Ge, Junrong Liang, Ping Chen, Jinchi Zhou, Tianyu Cao, Qi Wang, Xiaoliang Gao, Mingfu Tong, Sijun Hu, Yongzhan Nie, Daiming Fan, xin wang, Xiaodi Zhao, Yuanyuan Lu

**Affiliations:** 1 State Key Laboratory of Cancer Biology and National Clinical Research Center for Digestive Diseases, Xijing Hospital of Digestive Diseases, Fourth Military Medical University, 710032 Xi’an, China; 2 Department of Gastroenterology, Tangdu Hospital, Fourth Military Medical University, 710038 Xi’an, China; 3 The Affiliated Children’s Hospital of Xi’an Jiaotong University, 710003 Xi’an, China; 4 State Key Laboratory of Translational Medicine and Innovative Drug Development, Simcere Diagnostics Co., Ltd., 210042 Nanjing, China; 5 Department of Gastroenterology, Beijing Chao-Yang Hospital, Capital Medical University, 100020 Beijing, China; 6 National Institute of Biological Sciences, 102206 Beijing, China

**Keywords:** Colorectal cancer, Non-coding RNAs, Metastasis, Oncogenes

## Abstract

**Background:**

The small GTPase Ran is upregulated in multiple cancers and fundamental for cancer cell survival and progression, but its significance and molecular mechanisms in colorectal cancer (CRC) remain elusive.

**Methods:**

Ran expression was detected in CRC cell lines and tumour tissues. In vitro and in vivo functional assays were performed to examine the effects of Ran on cell proliferation and metastasis. The pathways and effectors regulated by Ran were explored by an unbiased screening. Bioinformatics prediction and experimental validation were used to identify the miRNA regulator for Ran.

**Results:**

Ran expression was frequently increased in metastatic CRC cells and tissues, especially in metastatic tissues. The upregulation of Ran correlated with poor CRC patient prognosis. Ran silencing reduced proliferation and metastasis of CRC cells both in vitro and in vivo. Ran regulated the expression of EGFR and activation of ERK and AKT signalling pathways. miR-802 was identified as an upstream regulator of Ran and miR-802 overexpression resulted in antiproliferative and antimetastatic activities.

**Conclusion:**

Our study demonstrates the oncogenic roles and underlying mechanisms of Ran in CRC and the novel miR-802/Ran/EGFR regulatory axis may provide potential biomarkers for the treatment of CRC.

## Background

Colorectal cancer (CRC), the third most commonly diagnosed cancer in males and the second in females worldwide, was responsible for 1.8 million new cases and an estimated 861,000 deaths globally in 2018.^1^ Despite the decline in CRC morbidity and mortality in recent years, there are still challenges for the treatment of advanced CRC patients whose prognoses are poor due to a late diagnosis and a lack of therapeutic options.^2^ Therefore, the need to discover novel biomarkers for early detection, prognosis prediction and therapeutic intervention in CRC is essential. We previously developed a CRC-specific monoclonal antibody, MC3, and further identified thioredoxin-like protein 2 (Txl-2) as its target.^3^ We also found that elevated Txl-2 was strongly correlated with histological grade and CRC patient prognosis and demonstrated that the overexpression of Txl-2 promoted cell invasion and metastasis through its interaction with the GTPase Ran.^4^ However, the roles and underlying mechanisms of Ran in the initiation and metastasis of CRC remain elusive.

Ran belongs to the Ras superfamily of GTPases, which act as molecular switches that are turned on by GTP binding and off by GTP hydrolysis to GDP.^5^ Ran has been shown to be involved in multiple cellular processes, including nucleocytoplasmic transport, assembly of the mitotic spindle, the regulation of cell cycle progression, and formation of the nuclear envelope.^6^ Emerging evidence has demonstrated that Ran is often expressed at high levels in various cancers and that these aberrant expression levels are associated with the increased aggressiveness of cancer cells and a poor patient prognosis.^7^ In pancreatic cancer, elevated Ran was found to promote proliferation and reduced apoptosis by regulating the expression of Survivin and cell cycle proteins.^8^ In ovarian cancer, overexpressed Ran was correlated with poor patient survival and enhanced cell invasion by increasing the membrane targeting and stabilisation of RhoA.^9^ We previously found that Ran is highly expressed in CRC tissues and positively correlated with tumour differentiation, local invasion and metastasis. The overall survival rate was significantly lower in CRC patients with Ran-positive tumours than in those with Ran-negative tumours.^10^ Although the expression patterns of Ran in several cancer types have been determined, the causes of its overexpression have not yet been fully explored.

MicroRNAs (miRNAs) are a kind of small noncoding RNA that can directly regulate gene expression at the posttranscriptional level by binding the 3’-untranslated region (3’-UTR) of target mRNAs.^11^ Genome-wide analyses demonstrated that miRNAs are frequently dysregulated in most cancer types, which may regulate the expression of important cancer-related genes.^12^ Recent studies have identified the critical roles of miRNAs in CRC initiation and metastasis and suggested that miRNA-based gene therapy provides a novel approach for CRC treatment.^13,14^ For instance, miR-143 acts as a suppressor of colorectal tumorigenesis by direct inhibition of KRAS translation.^15^ In addition, miR-34a-5p targets p53 and inhibits cell growth, migration and invasion in CRC cells in a p53-dependent manner.^16^ miR-500a-5p was also reported to target histone deacetylase 2 (HDAC2) and inhibit HDAC2-mediated proliferation in CRC cells.^17^ We previously showed that miR-302a inhibited CRC cell metastasis by targeting nuclear factor I B and restored cetuximab responsiveness by suppressing CD44.^18^ However, whether Ran is regulated by certain miRNAs during CRC initiation and progression remains unknown.

In this study, we found that Ran is significantly upregulated in CRC cells and tissues, especially in metastatic tissues, and that the upregulation of Ran correlates with poor CRC patient prognosis. Ran silencing reduced proliferation, induced apoptosis and inhibited the invasion and metastasis of CRC cells both in vitro and in vivo. Mechanistically, Ran regulated the expression of epidermal growth factor receptor (EGFR) and activation of extracellular signal-regulated kinase (ERK) and AKT signalling pathways. miR-802 was identified as an upstream regulator of Ran and miR-802 overexpression abrogated the oncogenic effects of Ran. Collectively, our results demonstrated Ran is an oncogene and uncovered its regulatory mechanism in CRC.

## Methods

### Tissue specimens

Two commercial CRC tissue microarrays (HLin-Ade075Met-01 and HCol-Ade060Lym-01) containing samples from 45 primary CRC tissues, 34 paired adjacent non-tumour tissues and 51 metastatic tissues were purchased from Outdo Biotech Company (Shanghai, China). Twelve primary CRC tissue samples and paired adjacent non-tumour tissue samples were collected from patients who had undergone CRC surgery at Xijing Hospital of Digestive Diseases; the collected samples were frozen in liquid nitrogen. All samples were clinically and pathologically shown to be correctly labelled. Informed consent was obtained from all patients, and this study was approved by Xijing Hospital’s Protection of Human Subjects Committee.

### Cell culture

The human CRC cell lines SW480, HT29, HCT-8, SW620, HCT116 and DLD-1 and the human normal colonic mucosal cell line NCM460 were obtained from the American Type Culture Collection (ATCC). KM12C and KM12SM were obtained from Robert Coffey laboratory (Vanderbilt University). All cells were cultured in Dulbecco’s modified Eagle’s medium (Gibco, NY, USA) supplemented with 10% foetal bovine serum (HyClone, UT, USA) and 1% penicillin–streptomycin (Gibco) and incubated at 37 °C with 5% CO_2_.

### Immunohistochemistry

The slides were first incubated on a 60 °C heating panel for 1 h, then deparaffinised, subjected to antigen retrieval and endogenous peroxidase inactivation and incubated with the following primary antibodies: mouse anti-human Ran (BD Biosciences, CA, USA) and mouse anti-human Ki-67 (Cell Signalling Technology, MA, USA). Then the sections were incubated with horseradish peroxidase (HRP)-conjugated goat anti-rabbit or goat anti-mouse secondary antibodies (ZSGB, Beijing, China). The target molecules were detected in situ with diaminobenzidine (ZSGB), and the sections were counterstained with haematoxylin–eosin (ZSGB). Expression was visualised and classified based on the intensity of staining and the percentage of positive cells. The intensity score was divided into four grades: negative (0), weak (1), moderate (2), and strong (3). The percentage score was also divided into four grades: ≤1% (0), 2–25% (1), 26–50% (2), 51–75% (3), and ≥75% (4). The histological score was calculated by the following formula: histological score = intensity score × percentage score. An overall score of 0–12 was graded as negative (−, score: 0), weak (+, score: 1–4), moderate (++, score: 5–8) or strong (+++, score: 9–12).

### Quantitative real-time PCR (qRT-PCR)

Total RNA was extracted from cultured cells or freshly collected human tissues with TRIzol Reagent (Invitrogen, CA, USA) and purified using a miRNAeasy Kit (Qiagen, MD, USA) in accordance with the manufacturer’s instructions. For mRNA detection, total RNA was reverse transcribed with a QuantiTect Reverse Transcription Kit (Qiagen), and the double-stranded cDNA was amplified by real-time PCR by using SYBR Premix Ex Taq (Takara, Dalian, China). For miRNA detection, total RNA was used for complementary DNA synthesis with a TaqMan miRNA Reverse Transcription Kit (Applied Biosystems, MA, USA). Real-time PCR was performed with the use of TaqMan Fast Advanced Master Mix (Applied Biosystems). PCR primers for miR-802 and U6 were designed and synthesised by RiBoBio (Guangzhou, China). PCR primers for Ran, EGFR and glyceraldehyde 3-phosphate dehydrogenase (GAPDH) were synthesised by TaKaRa. The PCR primers were as follows: Ran-forward 5′-AGCAGTGTTTGCTCCACCTTCATA-3′, Ran-reverse 5′-CTGCCATCCACTGATGTTCCA-3′; EGFR-forward 5′-TGATTGGGGATCTTGGAGTTTT-3′, EGFR-reverse 5′-CTTGTGGCTTGTGCTCCTTG-3′; and GAPDH-forward 5′-GCACCGTCAAGGCTGAGAAC-3′, GAPDH-reverse 5′-TGGTGAAGACGCCAGTGG A-3′. The qRT-PCR analysis was conducted on a LightCycler 480 system (Roche, Basel, Switzerland). GAPDH was used as an internal control. The 2^−ΔΔCt^ method was used to determine the relative expression level of RNA of each sample relative to the reference sample.

### Western blot analysis

Cells were lysed using 200 µl of RIPA lysis buffer (Beyotime, Shanghai, China) supplemented with a protease inhibitor cocktail (Roche) and phosphatase inhibitors (Roche) for 20 min, and the concentration was determined with a BCA Protein Assay Kit (Beyotime). Denatured proteins were fractionated by sodium dodecyl sulfate–polyacrylamide gel electrophoresis and transferred to nitrocellulose membranes. After being blocked with 10% skim milk in TBST (150 mM NaCl, 120 mM Tris-HCl, pH 7.4, and 0.05% Tween 20), the membranes were incubated with the primary and secondary HRP-conjugated antibodies. The antibodies used were as follows: mouse anti-human Ran (BD Biosciences); mouse anti-human β-actin (Sigma-Aldrich, Darmstadt, Germany), rabbit anti-human proliferating cell nuclear antigen (PCNA), rabbit anti-human pro-poly ADP-ribose polymerase (pro-PARP), mouse anti-human cleaved-PARP, rabbit anti-human pro-caspase 3, rabbit anti-human cleaved caspase 3, rabbit anti-human AKT, rabbit anti-human pAKT, rabbit anti-human ERK, rabbit anti-human pERK, an EMT Antibody Sampler Kit (Cell Signalling Technology); rabbit anti-human GAPDH (Proteintech, Hubei, China); and goat anti-rabbit or goat anti-mouse IgG (ZSGB). GAPDH and β-actin were used as internal controls. Blots were scanned by a Molecular Imager ChemiDox XRS+ Imaging System with the Image Lab software (Bio-Rad Laboratories, Shanghai, China).

### Plasmid construction

The plasmid of Ran was constructed by inserting the Ran cDNA into the pCMV6-Entry vector (OriGene Technologies, Rockville, MD, USA) at SgfI and MluI sites. To construct a luciferase reporter vector, the wild-type 3’-UTR of Ran was PCR-amplified using genomic DNA from HEK293T cells as template. The corresponding mutant constructs were created by mutating the seed region of the miR-802-binding site. Both wild-type and mutant 3’-UTRs were cloned downstream of the luciferase gene in the psiCHECK-2 Luciferase vector. The constructs were verified by sequencing.

### Transient transfection and lentivirus infection

Small interfering RNAs against Ran (siRan), mimics of miR-802, miR-140-5p, miR-142-3p and corresponding negative control oligonucleotides were designed and synthesised by GenePharma. Ran siRan#1 5′-CGUCAUUUGACUGGUGAAUTT-3′, Ran siRan#2 5′-GAG UUACUUACAAGAAUGUTT-3′ and the negative control siRNA 5′-UUCUCCGAACGUG UCACGUTT-3′ were transfected into HCT116 and DLD-1 cells, whereas HT-29 and SW480 cells were transfected with a miR-802 mimic and a scrambled negative control miRNA. Transfection of the siRNAs and miRNA mimics was performed using DharmaFECT transfection reagent (Thermo Fisher Scientific, MA, USA) according to the manufacturer’s instructions. The successful knockdown of Ran was confirmed by western blot, and the overexpression of miR-802 was confirmed by qRT-PCR. Lentivirus carrying the human *Ran* gene (LV-Ran) and short hairpin RNA targeting Ran (LV-shRan) were purchased from GeneChem (Shanghai, China). An empty lentiviral vector was used as negative control. At 48 h postinfection, CRC cells were screened with 2.5 μg/ml puromycin (MP Biomedicals, CA, USA) for 2 weeks.

### Cell proliferation and apoptosis assays

Treated cells were seeded at a density of 1 × 10^3^ per well on 96-well plates. After incubation for 24, 48, 72, 96 and 120 h, Cell Counting Kit 8 (CCK-8, Kumanoto, Japan) was utilised to examine cell proliferation according to the manufacturer’s instructions. The absorbance was measured at 450 nm using a microplate reader (Thermo Fisher Scientific). An Annexin V-fluorescein isothiocyanate (FITC) Apoptosis Detection Kit (Sigma-Aldrich) was used for apoptosis assays. Cells staining positively for Annexin V-FITC and negatively for propidium iodide at 48 h after transfection were sorted using a fluorescence-activated cell sorter (BD Biosciences).

### In vitro migration and invasion assays

The cell migration and invasion capabilities were assessed by Transwell assay with an 8-µm pore, 24-well Transwell plates (Corning, NY, USA). For the migration assays, 2 × 10^5^ HT29 LV-Ran, SW480 LV-Ran, HCT116 LV-shRan, DLD-1 LV-shRan or corresponding negative control cells were seeded in the top chamber lined with an uncoated membrane (Millipore, MA, USA). In the invasion experiment, 2 × 10^5^ of the aforementioned cells were plated in the upper chamber coated with Matrigel (BD Biosciences). After incubation at 37 °C with 5% CO_2_ for an appropriate duration, cells that had migrated or invaded through the membrane were stained with 0.1% crystal violet and counted under a microscope (Olympus, Tokyo, Japan) to determine their relative numbers.

### Wound-healing assays

Wound-healing assays were performed according to the Culture-Insert 2 Well (Ibidi, Martinsried, Germany) manufacturer’s protocol. HT29 LV-Ran, SW480 LV-Ran, HCT116 LV-shRan, DLD-1 LV-shRan or corresponding negative control cells were diluted to a concentration of 5–7 × 10^5^ cells/ml, and 70 µl of the cell suspension was added to each well. The well was gently removed when the confluence reached 90–100%, and a scratch was made. The gap was imaged at 0 and 24 h after scratching by an Olympus camera.

### In vivo tumorigenicity and metastasis assays

Four-to-6-week-old female nude mice were cared for and maintained in the Experimental Animal Center of the Fourth Military Medical University. The Committee on the Use of Live Animals in Teaching and Research of the Fourth Military Medical University approved all animal experiments. Mice were housed in specific pathogen-free conditions and with a 12 h-day/night cycle with lights on at 8:00 a.m. in a temperature (27 ± 1 °C) and humidity (50 ± 10%) controlled room. All mice were allowed free access to water and a balanced diet and were free of all viral, bacterial and parasitic pathogens. All mice were randomised into each group by picking random numbers. All mice were killed in their individually ventilated cages with carbon dioxide (25% chamber volume per minute).

Luciferase-tagged SW480 LV-Ran, HCT116 LV-shRan and corresponding negative control cells were used in subcutaneous xenograft and lung metastasis assays. For in vivo tumorigenicity assays, 1 × 10^6^ of the abovementioned cells in 150 µl of phosphate-buffered saline (PBS) were subcutaneously injected into the flanks of mice (*n* = 5 per group). The tumour volumes and bioluminescence were measured every 3 days. Mice were killed at 4 weeks, and the subcutaneous tumours were dissected for further histological examination. For in vivo metastasis assays, 1 × 10^6^ of the abovementioned cells were suspended in 100 µl of PBS and injected into the tail veins of nude mice (*n* = 5 per group). Five weeks after injection, bioluminescence was detected every week. The survival duration of the mice was monitored. Eight weeks after injection, mice were killed, and the lung metastases were dissected for further histological examination. In order to conduct bioluminescence imaging (BLI), all mice were under inhalation anaesthesia (2–3% isoflurane mixed with 30% oxygen and 70% nitrous oxide) and received intraperitoneal injection of D-luciferin (150 mg/kg). BLI was detected with an IVIS 100 Imaging System (PerkinElmer, MA, USA).

### Luciferase reporter assay

The indicated cells cultured in 24-well plates were co-transfected with the miR-802 mimic or negative control and the indicated wild-type or mutant psiCHECK2-3’UTR plasmids using Lipofectamine 2000 (Thermo Fisher Scientific). Cells were collected and lysed for luciferase assays 48 h after transfection. A Dual-Luciferase Assay (Promega, WI, USA) was used to quantify the luciferase activity in accordance with the manufacturer’s instructions. *Renilla* luciferase activity was normalised to firefly activity and presented as the relative luciferase activity. All assays were performed in triplicate.

### Databases

The Cancer Genome Atlas (TCGA; https://cancergenome.nih.gov) data sets were used to determine the expression of Ran mRNA and miR-802 in human cancer specimens and normal tissues.

### Statistical analysis

Data collected from at least three independent experiments were analysed using the SPSS 17.0 (SPSS Inc., USA) software. Normally distributed data and non-normally distributed data are presented as the mean ± SD, and Student’s unpaired *t* test and Mann–Whitney *U* test were used for comparisons between two groups, respectively. Classification data were analysed using Fisher’s exact test. Frequencies of categorical variables were compared using χ^2^ test. Spearman’s rank correlation coefficients were calculated for assessing mutual association among clinical results. *P* < 0.05 was considered representative of a significant difference (*P < 0.05, **P < 0.01 and ***P < 0.001).

## Results

### Ran expression is significantly upregulated in human CRC cells and tissues

**Fig. 1:**
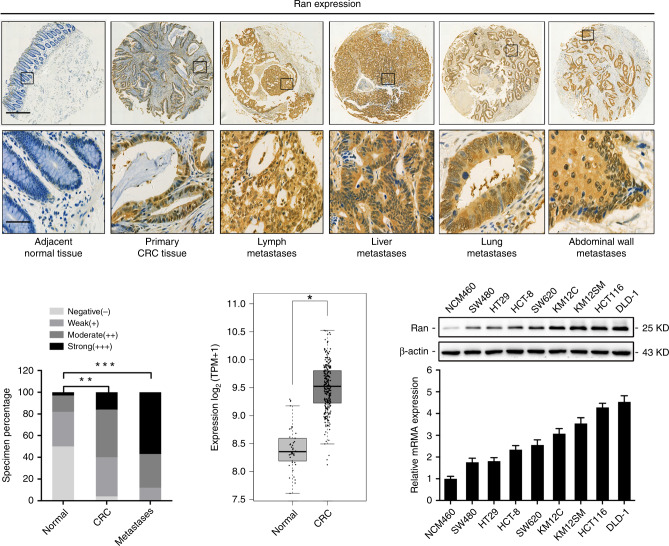
Ran is upregulated in CRC tissues and cells. **a** Representative images of Ran expression in adjacent non-tumour tissues, primary CRC tissues lymph metastases, liver metastases, lung metastases and abdominal wall metastases detected by IHC staining. Scale bars: 600 μm (upper) and 40 μm (lower). **b** IHC score grades of Ran expression in normal tissues (*n* = 34), primary CRC tissues (*n* = 45) and metastatic tissues (*n* = 51) of CRC patients (**P < 0.01 and ***P < 0.001). **c** The TCGA expression data for Ran in normal tissues and primary CRC tissues. **d** Western blot (above) and qRT-PCR (below) analysis of Ran expression in a panel of CRC cell lines (*P < 0.05).

To determine the expression pattern of Ran in CRC, immunohistochemistry (IHC) was employed to examine its expression in tissue microarrays containing 45 primary CRC tissues, 34 adjacent non-tumour tissues and 51 metastatic tissues. We found that the Ran staining intensity was significantly increased in primary CRC tissues compared with adjacent tissues, and Ran was expressed at higher levels in metastatic tissues than in primary CRC tissues (Fig. 1a, b). Correlation analyses revealed that Ran expression was positively correlated with CRC metastasis status, tumour stage, histological grade and poor patient prognosis (Table 1). Furthermore, we investigated the expression of Ran in the TCGA data set and found that the expression of Ran was significantly increased in CRC tissues compared to normal tissues (Fig. 1c). In addition, we measured the levels of Ran in a panel of CRC cell lines and an immortalised colonic epithelial cell line (NCM460). Western blot and qRT-PCR analyses showed that the expression of Ran was upregulated in the CRC cell lines compared with the epithelial cell line at both the protein and mRNA levels (Fig. 1d, e). Taken together, these results suggest that Ran may play important roles in driving CRC development and progression.

**Table 1:** Correlation of Ran expression with patient clinicopathological variables in CRC tissues.

Variables	All cases (*n* = 45)	Expression of Ran				*P* values
		−	+	++	+++	
Gender						0.669
Female	17	1	5	8	3	
Male	28	1	11	12	4	
Age (years)						0.118
≤65	32	2	13	13	4	
>65	13	0	3	7	3	
Tumour size (cm)						0.040
≤5	25	1	6	12	6	
>5	20	1	10	8	1	
Differentiation						0.023
High	5	1	3	1	0	
Moderate	30	1	11	15	3	
Poor	10	0	2	4	4	
Metastasis						0.016
No	17	2	9	4	2	
Yes	28	0	7	16	5	

### Ran sustains CRC cell proliferation and apoptosis evasion in vitro and in vivo

**Fig. 2:**
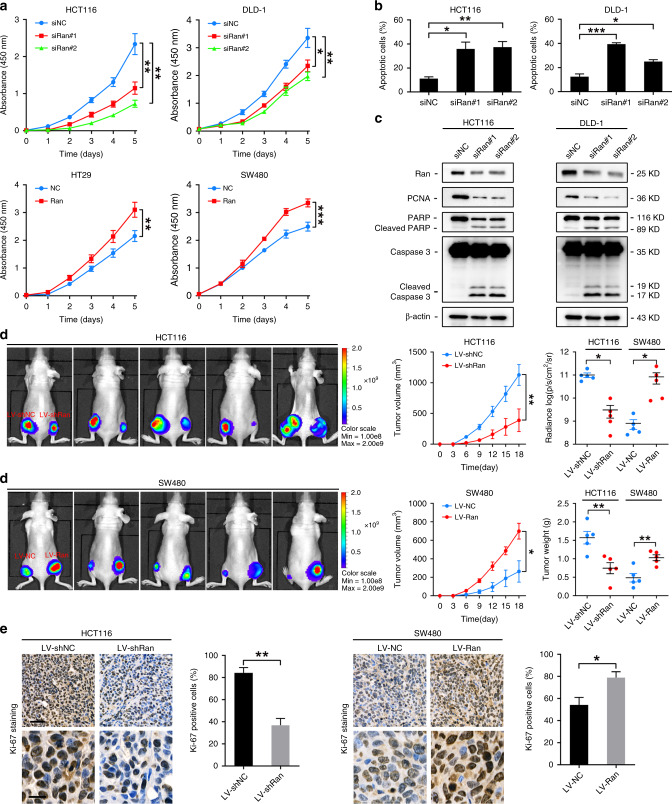
Ran promotes CRC cell proliferation in vitro and in vivo. **a** The cell proliferation capacity of Ran-knockdown HCT116 and DLD-1 cells (above) and Ran-overexpressing HT29 and SW480 cells (below), as determined by CCK-8 assay (*P < 0.05, **P < 0.01 and ***P < 0.001). **b** Apoptosis rate of Ran-knockdown HCT116 and DLD-1 cells and corresponding control cells based on fluorescence-activated cell sorting (*P < 0.05, **P < 0.01 and ***P < 0.001). **c** Western blot analysis of the expression of apoptotic markers in Ran-knockdown cell models. **d** Left: representative bioluminescence images of the tumours in nude mice are shown at 4 weeks after subcutaneous injection of Ran-knockdown HCT116 cells (above) or Ran-overexpressing SW480 cells (below) and their controls (*n* = 5 mice per group). Right: the tumour volume was measured every 3 days (middle). The radiance, volumes and weights of tumours were collected and compared in Ran-knockdown and Ran-overexpression models (*P < 0.05, **P < 0.01). **e** Ki-67 staining in xenograft tumour tissues and the percentages of Ki-67-positive cells were measured. Scale bars: 50 μm (above) and 20 μm (below); *P < 0.05 and **P < 0.01.

To investigate the role of Ran in CRC, we established gain- and loss-of-function cell models by transfecting CRC cells with a plasmid expressing Ran or siRNA oligos targeting Ran (siRan), respectively. Ran silencing and overexpression were confirmed by western blot (Fig. S1a, b). CCK-8 assay results indicated that Ran knockdown significantly inhibited HCT116 and DLD-1 cell proliferation (Fig. 2a). In contrast, the overexpression of Ran accelerated growth in HT29 and SW480 cells (Fig. 2a). In addition, knocking down Ran increased the apoptosis rate and induced the expression of the apoptotic markers cleaved caspase 3 and PARP, whereas it decreased the expression of the proliferation marker PCNA (Fig. 2b, c). To further determine whether Ran induces tumorigenesis in vivo, we infected HCT116 cells with lentivirus expressing Ran (LV-Ran) and SW480 cells with lentivirus expressing Ran shRNA (LV-shRan). HCT116-LV-shRan, SW480-LV-Ran and the corresponding control cells were subcutaneously injected into the flanks of nude mice to establish xenograft tumours. The tumour volumes were monitored, and the growth curves of the tumours were plotted accordingly. The tumour size and weight were significantly reduced in mice implanted with Ran-silenced HCT116 cells but increased in mice implanted with Ran-overexpressing SW480 cells (Fig. 2d). IHC staining showed that, compared with that in the control group, the Ki-67 staining intensity was reduced in xenografts from the Ran-silenced group and increased in the xenografts from the Ran-overexpression group (Fig. 2e). Taken together, these results demonstrate that Ran promotes CRC cell proliferation and reduces CRC apoptosis both in vitro and in vivo.

### Ran enhances CRC cell migration, invasion and metastasis

**Fig. 3:**
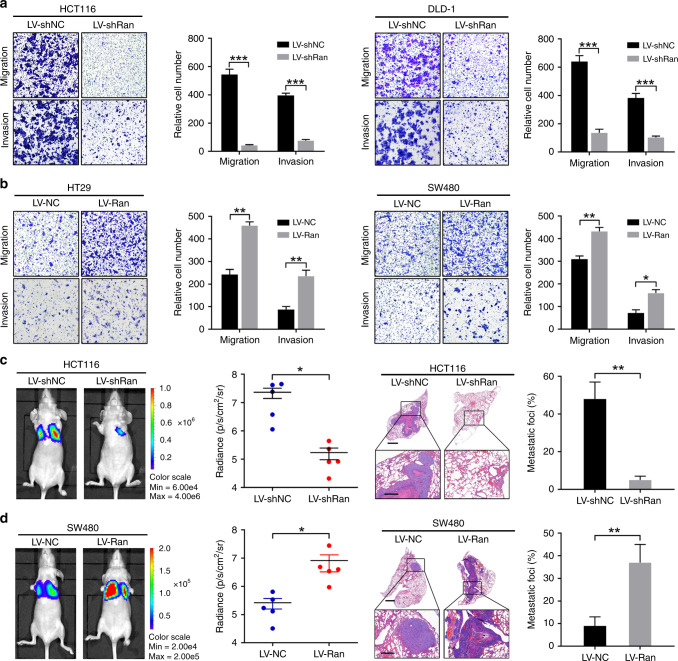
Ran enhances CRC cell migration, invasion and metastasis. **a**, **b** Transwell assays and corresponding data analyses showing the migration and invasion abilities of HCT116 and DLD-1 cells infected with LV-shRan or negative controls (**a**) and SW480 and HT29 cells infected with LV-Ran or negative controls (**b**). **c**, **d** Representative bioluminescence images of tumours in nude mice are shown at 8 weeks after tail vein injection of Ran-knockdown HCT116 cells (**c**, left), Ran-overexpressing SW480 cells (**d**, left) and the respective control cells (*n* = 5 mice per group). The radiance, H&E staining of lung tissues and percentage of metastatic foci from the different groups are shown. Scale bars: top, 1000 μm; bottom, 200 μm (*P < 0.05, **P < 0.01, ***P < 0.001).

To further examine whether Ran affects tumour migration and invasion, we performed Transwell migration and invasion assays with CRC cells infected with LV-Ran or LV-shRan. Upon Ran expression downregulation, the migration and invasion of HCT116 and DLD-1 cells were markedly decreased (Fig. 3a). Conversely, Ran expression upregulation enhanced HT-29 and SW480 cell migration and invasion (Fig. 3b). Wound-healing assays also indicated that Ran knockdown inhibited CRC cell migration (Fig. S1c). To determine the effect of Ran on metastasis, CRC cells with stably silenced or overexpressed Ran, as well as their negative controls, were delivered into nude mice by tail vein injection. After 6 weeks, in vivo BLI showed that the luminescence intensities in the lungs were substantially lower in mice in the HCT116-LV-shRan cell group than mice in the control group (Fig. 3c). The number of metastatic nodules in the lungs were decreased in HCT116-LV-shRan cell group (Fig. 3c). Consistently, the luminescence intensities and the number of metastatic nodules in the lungs were increased after Ran overexpression (Fig. 3d). Taken together, these results indicate that Ran promotes CRC migration, invasion and metastasis in vitro and in vivo.

### Ran regulates EGFR-mediated ERK and AKT signalling in CRC cells

**Fig. 4:**
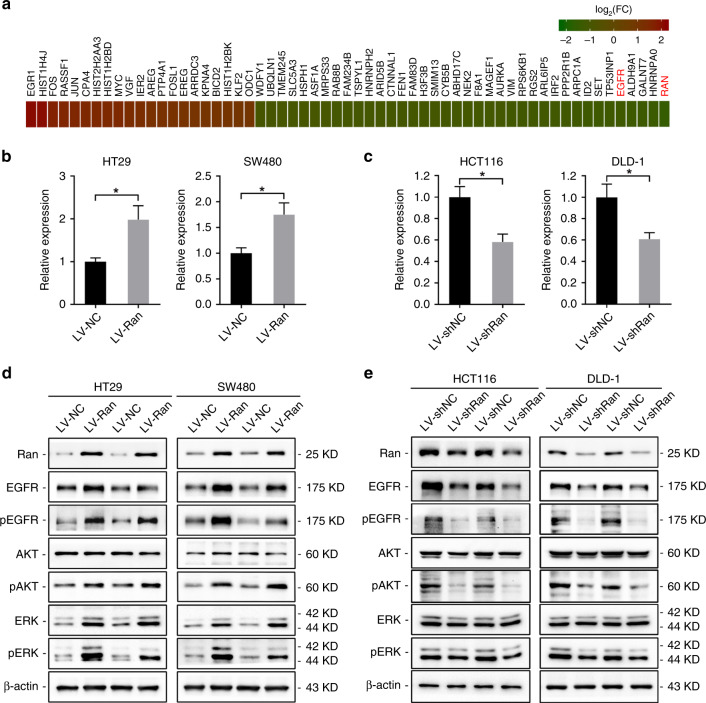
Ran regulates malignancy phenotypes in CRC cells through EGFR-dependent signalling. **a** Heatmap showing 38 genes with downregulated expression and 21 genes with upregulated expression at the mRNA level after Ran knockdown in HCT116 cells. The cut-off value was a twofold expression change, and the scale bar shows colour-coded differences in expression from the mean. **b**, **c** EGFR expression in SW480 and HT29 cells infected with LV-Ran or negative controls (**b**) and HCT116 and DLD-1 cells infected with LV-shRan or negative controls (**c**) was detected by qRT-PCR (*P < 0.05). **d**, **e** Western blot was used to detect the expression of ERK and AKT signalling pathway-related proteins in HT29 and SW480 cells infected with LV-Ran or negative controls (**d**) and HCT116 and DLD-1 cells infected with LV-shRan or negative controls (**e**).

To investigate the underlying mechanism by which Ran drives CRC development and progression, we performed gene expression microarray profiling to identify potential Ran targets. Using twofold as a cut-off value for differentially expressed genes (DEGs), we found that the expression of 38 genes was downregulated and of 21 genes was upregulated at the mRNA level after Ran knockdown (Fig. 4a). Among these DEGs, EGFR was selected for further investigation because it plays important roles in the regulation of several pathways related to tumour growth and metastasis. qRT-PCR confirmed that EGFR expression was decreased after Ran knockdown and that Ran overexpression increased EGFR levels (Fig. 4b. c). We found that knockdown of Ran expression decreased the total level of EGFR, as well as the phosphorylation levels of EGFR, AKT and ERK1/2, in HCT116 and DLD-1 cells (Fig. 4d). IHC staining also confirmed that expression of EGFR, pAKT and pERK were all decreased or lost in the Ran-silenced xenografts (Fig. S2). On the contrary, the expression levels of the total and phosphorylated forms of EGFR, AKT and ERK1/2 were significantly increased when Ran was overexpressed in HT29 and SW480 cells (Fig. 4e). Taken together, these results suggest that Ran promotes malignancy phenotypes in CRC cells by regulating EGFR expression and activating ERK and AKT signalling.

### miR-802 directly targets Ran in CRC cells

**Fig. 5:**
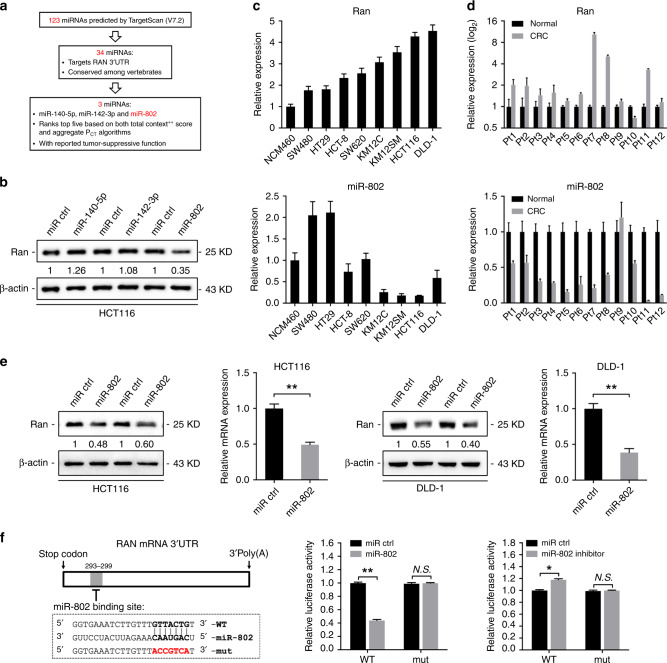
miR-802 downregulates Ran expression by directly binding its 3’-UTR. **a** Schematic diagram of miR-802 identification. Bioinformatics algorithms were employed to identify potential regulators. We used the TargetScan miRNA database to computationally predict 123 potential miRNAs, 34 of which target the Ran 3’-UTR and are conserved among vertebrates. Among these 34 miRNAs, we focussed on miR-802, miR-40-5p and miR-142-3p because they rank among the top five in both the total context ++ score and aggregate *P*_CT_ algorithms and might play a negative role in tumorigenesis according to previous studies. **b** Western blot analysis of Ran expression in HCT116 cells transfected with miR-802, miR-40-5p or miR-142-3p mimics and their controls. **c** qRT-PCR analysis of Ran expression (above) and miR-802 expression (below) in a panel of CRC cells. **d** Ran expression (above) and miR-802 expression (below) in 12 cases of primary CRC tissues and paired normal tissues detected by qRT-PCR. **e** Western blot and qRT-PCR analysis of Ran expression in HCT116 (left) and DLD-1 (right) cells transfected with miR-802 mimic or negative control and in HCT116 cells transfected with miR-802 mimic or negative control (**P < 0.01). **f** Left: a schematic representation of the predicted duplex sequences between the Ran 3’-UTR and miR-802. Mutations were designed at the predicted miR-802-binding sites (middle and right). The Ran wild-type and mutant reporter plasmids were co-transfected with a miR-802 mimic (middle), miR-802 inhibitor (right) or the respective controls. Luciferase activity values were measured and analysed (n.s.: not significant; *P < 0.05, **P < 0.01).

To investigate the Ran regulatory mechanism at the miRNA level, a bioinformatics strategy was employed to identify potential miRNAs targeting Ran (Fig. 5a). Briefly, we identified 123 miRNAs that were predicted to target the 3’-UTR of Ran by using the miRNA database TargetScan.^19^ Among them, 34 miRNAs were highly conserved among vertebrates. We found that 3 of the 34 miRNAs (miR-140-5p, miR-142-3p and miR-802) that ranked among the top 5 calculated by both the total context ++ score and aggregate *P*_CT_ algorithms and are reported to be involved in tumorigenesis. Mimics of the three miRNAs were then transfected into HCT116 cells, and western blot revealed that transfection of the miR-802 mimic reduced the expression of Ran (Fig. 5b). We then detected the expression levels of miR-802 and Ran in a panel of CRC cell lines by qRT-PCR and found that the endogenous Ran and miR-802 levels were inversely correlated (Fig. 5c). We further validated the expression of Ran and miR-802 in 12 paired primary CRC tissues and adjacent non-tumour tissues. qRT-PCR analysis showed that Ran expression was increased in 11 of the 12 primary CRC tissues. However, miR-802 was partially or totally lost in the 11 CRC tissues and its expression was inversely correlated with that of Ran (Fig. 5c). Furthermore, western blot analyses showed that the overexpression of miR-802 suppressed Ran expression in HCT116 and DLD-1 cells (Fig. 5e). To assess whether miR-802 directly binds to the 3’-UTR of Ran, we performed dual-luciferase reporter assays in 293T cells. miR-802 overexpression suppressed the luciferase activity of the Ran 3’-UTR reporter wild-type construct, whereas this effect was abolished by the introduction of a mutation into the miR-802-binding sequences in the mutant construct (Fig. 5f). Taken together, these results indicate that miR-802 downregulates Ran expression by directly targeting its 3’-UTR.

### miR-802 inhibits CRC cell proliferation and invasion by suppressing Ran

**Fig. 6:**
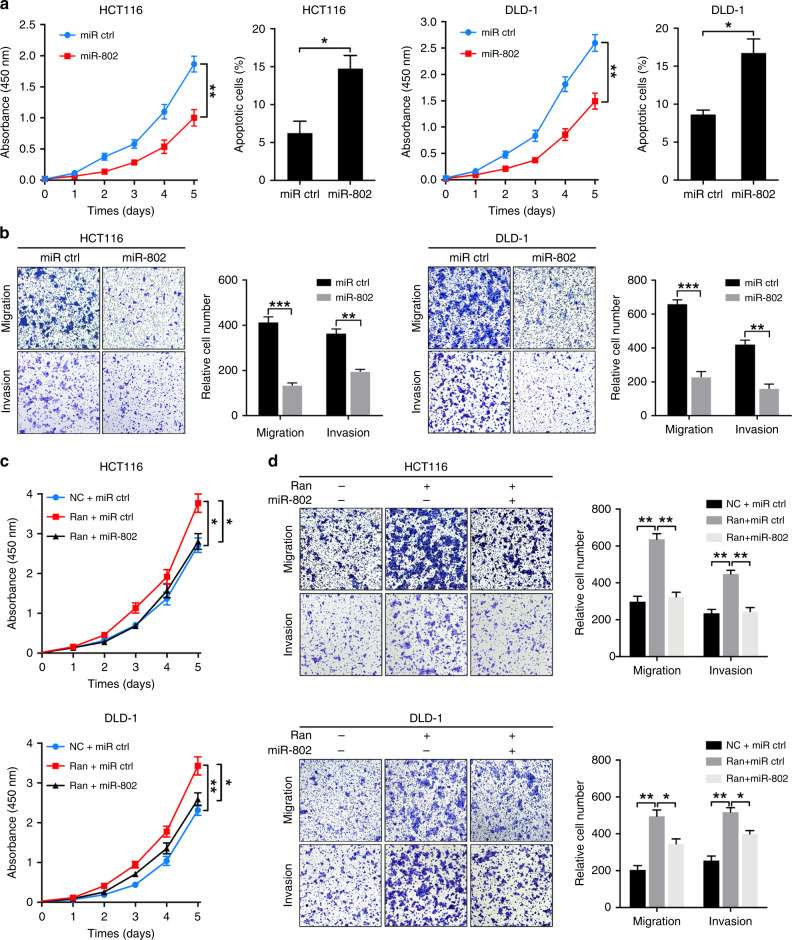
miR-802 inhibits CRC growth and metastasis by suppressing Ran. **a** Cell proliferation and apoptosis assays of HCT116 cells (left) and DLD-1 (right) cells transfected with a miR-802 mimic or corresponding negative control (*P < 0.05). **b** Transwell assays and corresponding data analyses showing the migration and invasion abilities of HCT116 (left) and DLD-1 (right) cells transfected with miR-802 mimic or corresponding negative control (**P < 0.01 and ***P < 0.001). **c**, **d** HCT116 cells and DLD-1 cells were transfected with LV-Ran or a negative control, along with miR-802 mimic or corresponding control. The proliferation of pretreated HCT116 cells and DLD-1 cells was determined by CCK-8 assay (**c**). Cell migration and invasion abilities were detected by Transwell assay (**d**) (*P < 0.05, **P < 0.01).

To investigate the effects of miR-802 on CRC growth and metastasis, we transfected the miR-802 mimic into DLD-1 and HCT116 cells. Upon miR-802 overexpression, the proliferation and antiapoptotic capabilities of HCT116 and DLD-1 cells were significantly decreased (Fig. 6a). Consistently, Transwell assays revealed that miR-802 overexpression suppressed the migration and invasion of DLD-1 and HCT116 cells (Fig. 6b). We then speculated that the suppressive effects of miR-802 in CRC cells are mediated by targeting Ran. To this end, we co-transfected a Ran expression plasmid with its 3’-UTR and miR-802 into CRC cells. As expected, the increased proliferation, migration and invasion capabilities were partially antagonised by the ectopic expression of miR-802 (Fig. 6c, d). Taken together, these results suggest that miR-802 inhibits proliferation and metastasis by targeting Ran in CRC cells.

## Discussion

In the present study, we found that Ran expression was significantly upregulated in primary and metastatic CRC tissues compared with adjacent tissues. In vitro and in vivo experiments demonstrated that Ran promoted the proliferation, migration, invasion and metastasis of CRC cells. Mechanistically, we identified a novel miR-802/Ran/EGFR regulatory axis that may contribute to the development and progression of CRC.

The dysregulation of Ran plays a significant role in the process of initiation and metastasis in various cancer types; however, its underlying mechanisms are not fully understood.^20,21^ In the present study, we identified EGFR as a regulatory target of Ran using an unbiased gene expression microarray profiling method. EGFR is a transmembrane protein with cytoplasmic kinase activity that transduces specific growth factor signalling from the extracellular space into cells.^22^ Inappropriate activation of EGFR contributes to tumorigenesis in multiple solid tumour types and combines to provide oncogenic signals to cancer cells.^23,24^ Aberrant EGFR signals overactivate downstream pro-oncogenic signalling pathways.^25^ Our study showed that the knockdown of Ran significantly reduced not only both the total and phosphorylated forms of EGFR but also the expression of phosphorylated ERK and AKT, suggesting that Ran promotes malignancy phenotypes in CRC cells by regulating the expression of EGFR and activation of ERK and AKT signalling. Our observation is consistent with a previous study showing that the downregulation of Ran led to reduced activation of the phosphoinositide-3 kinase/AKT/mammalian target of rapamycin C1 and Ras/MEK (mitogen-activated protein/ERK kinase)/ERK pathways.^26^ How the aberrant expression of Ran affects the activity of the EGFR-mediated ERK and AKT signalling pathways is not largely understood. One explanation might be that Ran, based on its role as a shuttle protein, mediates the nuclear–cytoplasmic translocation of EGFR. Nevertheless, the interaction between Ran and EGFR remain to be further investigated.

The causes of the aberrant overexpression of Ran in cancers remain unclear. miRNAs are important epigenetic regulators that modulate the expression of critical cancer-related genes and thereby function as oncogenes or tumour-suppressor genes.^27^ To date, few studies have reported specific miRNAs that regulate the expression of Ran. In oesophageal cancer, miR-203 suppresses tumour growth and invasion by repressing Ran expression.^28^ In rhabdomyosarcoma cells, miR-197 targets Ran and modulate internal ribosome entry site activity, which decreased viral replication.^29^ In the present study, we employed bioinformatics analyses and subsequent experiments to identify the regulatory miRNA of Ran in CRC cells. We found that miR-802 was predicted to target Ran by bioinformatics algorithms and observed an inverse correlation between miR-802 and Ran in CRC cell lines and tissue samples. Furthermore, miR-802 was found to inhibit the Ran protein level as well as its mRNA level, indicating its posttranscriptional repressive effect on Ran expression. Using a luciferase reporter gene assay, we demonstrated that miR-802 inhibited Ran by directly binding its 3’-UTR. Since miRNAs are regarded as new biomarkers and promising therapeutic targets for cancer treatment, whether other miRNAs regulate Ran expression remains to be explored.

One single miRNA can affect the translation of multiple genes and may lead to profound phenotypic responses in different cell contexts.^11^ The aberrant expression of miR-802 has been reported in various cancers and shown to regulate multiple malignant phenotypes. For instance, in prostate cancer, miR-802 acts as a tumour suppressor by repressing flotillin-2 expression.^30^ In gastric cancer, miR-802 suppresses the migration and invasion of gastric cancer cells by targeting RAB23.^31^ In breast cancer, miR-802 suppresses breast cancer proliferation by regulating FoxM1 expression.^32^ However, conflicting evidence for the role of miR-802 in certain cancers has also been reported. In osteosarcoma, miR-802 has been shown to play an oncogenic role in cell proliferation by decreasing the expression of p27.^33^ This finding can be partially explained by the theory that the binding of miRNAs to mRNAs is achieved with imperfect complementarity and because the expression levels of miRNA targets vary in different cell and tissue types. In the present study, we provide several lines of evidence to support the notion that miR-802 functions as a tumour-suppressive miRNA by targeting Ran in CRC. Overexpression of miR-802 inhibited proliferation and invasion but induced apoptosis in CRC cells. Importantly, the increased proliferative and metastatic abilities conferred by Ran overexpression were partially abrogated by ectopic miR-802 expression. Collectively, our results indicate that Ran is a direct and functional target of miR-802 and suggest miR-802 as a potential therapeutic agent to target Ran.

In summary, our study reveals the oncogenic roles of Ran in promoting CRC proliferation and metastasis and uncovers the functional mechanisms by which Ran regulates EGFR expression and its downstream ERK and AKT pathways. In addition, Ran was identified as a genuine target of miR-802 that could suppress Ran-mediated proliferation, migration and invasion in CRC cells. The miR-802/Ran/EGFR axis represents a novel component of the mechanism underlying CRC progression and may provide potential biomarkers and therapeutic targets against CRC.

## Supplementary information

Supplementary materials

## Data Availability

Summarised primary research data are presented in the paper. No publicly available data set has been generated as part of this work.
