# Supplementary material for: Regulation of the small GTPase Ran by miR-802 modulates proliferation and metastasis in colorectal cancer cells
Source: Br J Cancer. 2020 Mar 25;122(11):1695–706. doi: 10.1038/s41416-020-0809-7 (PMC7250854; doi:10.1038/s41416-020-0809-7)
Supplement: Supplementary file 1 — Supplementary materials [file 41416_2020_809_MOESM1_ESM.pdf]

Supplementary Figure Legends

Figure S1 (a, b) siRNAs against Ran (siRan) were used to knock down Ran in HCT116 and DLD-1 cells, and a lentiviral Ran expression vector was used to stably transduce HT29 and SW480 cells. The silencing and overexpression of Ran were confirmed by Western blot. (c) Wound-healing assays were used to detect the migration abilities of Ran-knockdown and Ran-overexpressing CRC cells (*$P<0.05$, **$P<0.01$).

Figure S2 EGFR, pAKT and pERK staining in xenograft tumour tissues and the percentages of positive cells were measured. Scale bars: 50 μm (above) and 20 μm (below); **$P<0.01$.

Figure S1

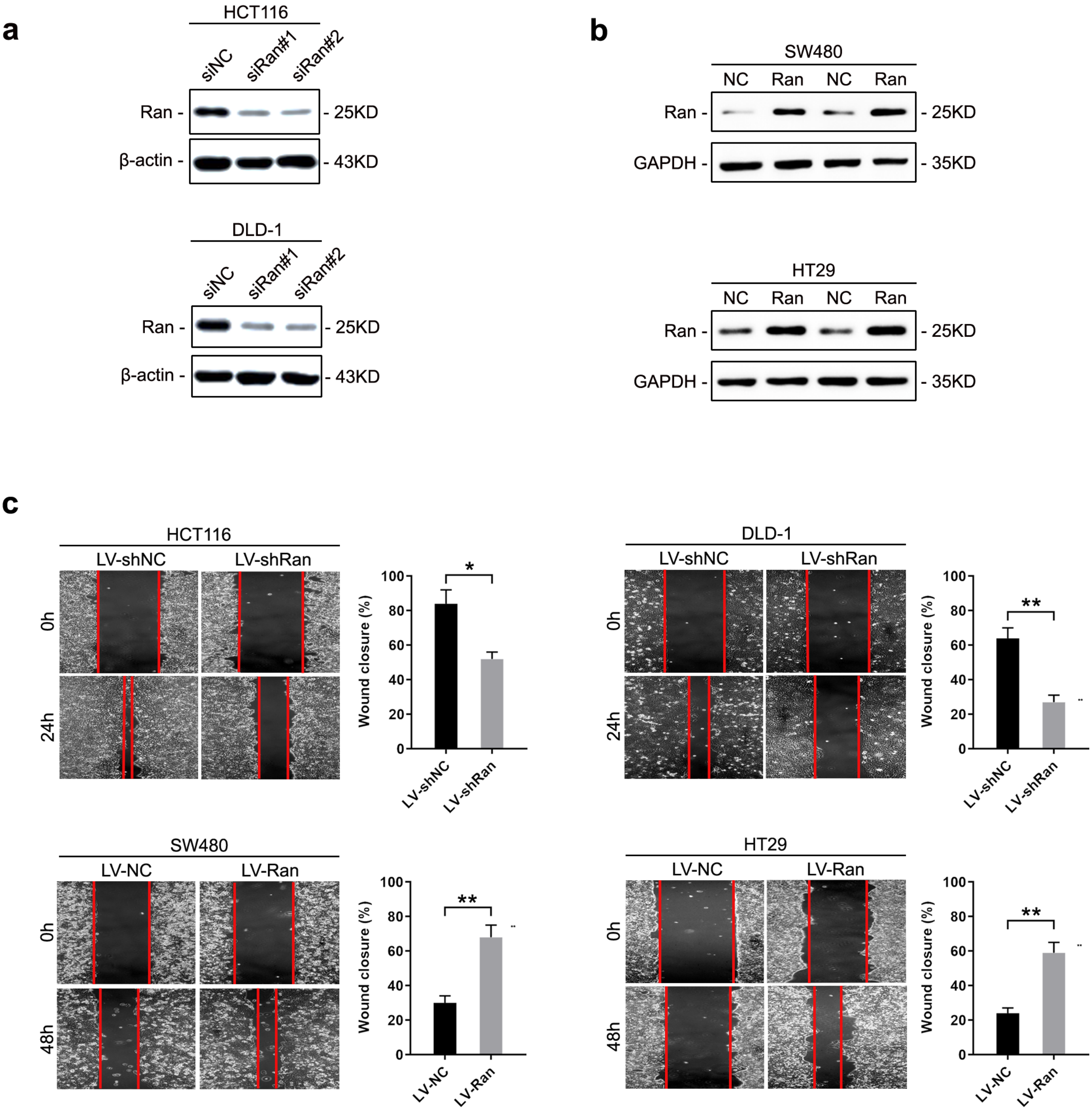

a
HCT116
siNC
siRan#1
siRan#2
Ran -
- 25KD
β-actin -
- 43KD
DLD-1
siNC
siRan#1
siRan#2
Ran -
- 25KD
β-actin -
- 43KD
b
SW480
NC
Ran
NC
Ran
Ran -
- 25KD
GAPDH -
- 35KD
HT29
NC
Ran
NC
Ran
Ran -
- 25KD
GAPDH -
- 35KD
c
HCT116
LV-shNC
LV-shRan
0h
24h
Wound closure (%)
LV-shNC
LV-shRan
*
DLD-1
LV-shNC
LV-shRan
0h
24h
Wound closure (%)
LV-shNC
LV-shRan
**
SW480
LV-NC
LV-Ran
0h
48h
Wound closure (%)
LV-NC
LV-Ran
**
HT29
LV-NC
LV-Ran
0h
48h
Wound closure (%)
LV-NC
LV-Ran
**

Figure S2

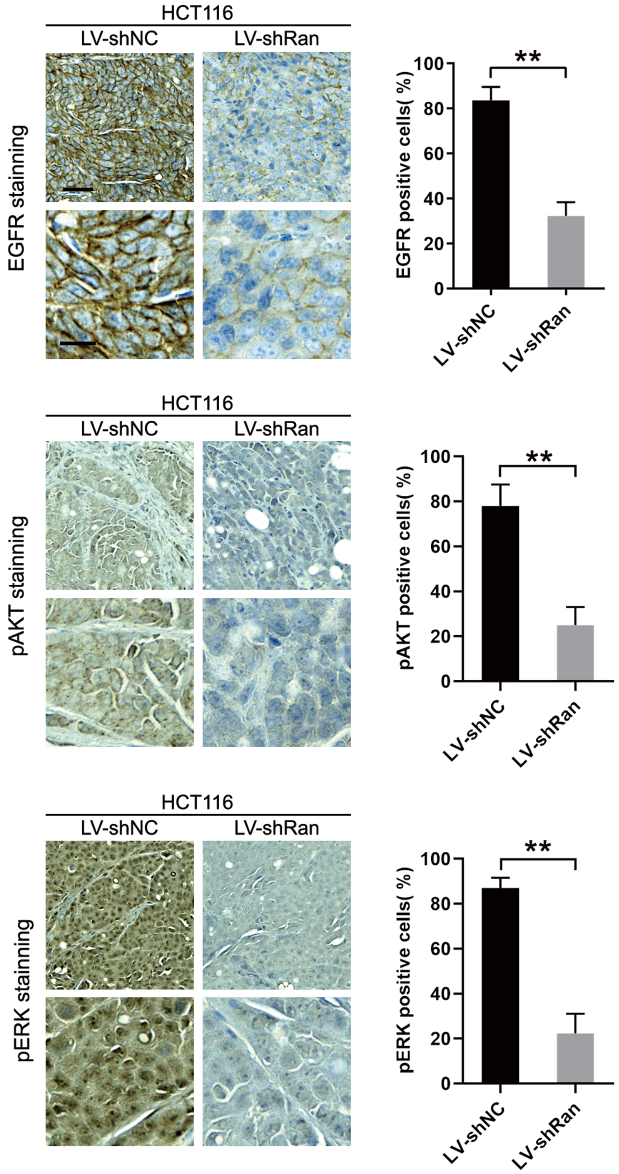

HCT116
LV-shNC
LV-shRan
EGFR stainning
EGFR positive cells(%)
100
80
60
40
20
0
**
LV-shNC
LV-shRan
HCT116
LV-shNC
LV-shRan
pAKT stainning
pAKT positive cells(%)
100
80
60
40
20
0
**
LV-shNC
LV-shRan
HCT116
LV-shNC
LV-shRan
pERK stainning
pERK positive cells(%)
100
80
60
40
20
0
**
LV-shNC
LV-shRan
